# Supplementary material for: The transcription factor Dof3.6/OBP3 regulates iron homeostasis in Arabidopsis
Source: EMBO J. 2024 Nov 13;44(1):251–68. doi: 10.1038/s44318-024-00304-0 (PMC11696086; doi:10.1038/s44318-024-00304-0)
Supplement: Supplementary file 1 — Appendix [file 44318_2024_304_MOESM1_ESM.pdf]

Appendix for

The transcription factor Dof3.6/OBP3 regulates iron homeostasis in Arabidopsis

Table of contents

Appendix Figure S1: Alignment analysis of Arabidopsis OBP subgroup proteins........................2

Appendix Figure S2: qPCR analysis of bHLH1b genes expression level.....................................3

Appendix Figure S3: Analysis of root phenotype and expression level of *FER* genes in plants with constitutive overexpression of *OBP3*..4

Appendix Figure S4: The gene expression levels of FRO2 and IRT1 and the activity of ferric-chelate reductase in Col-0 and *obp3* mutant root..5

Appendix Figure S5: Relative expression levels of iron deficiency related genes in Col-0 WT and *obp3-2* mutant..6

Appendix Figure S6: Codon optimization and *E. coli* bacterial expression of OBP3 protein........7

Appendix Figure S7: The *bHLH38*, *bHLH39*, *bHLH101* expression levels in Col-0, *obp3* single and *obp3ilr3* double mutant..8

Appendix Figure S8: The direct interaction between OBP3 and BTS was also conducted by GST pull-down assay..9

Appendix Figure S9: BTS1 promotes OBP3 degradation via the 26S proteasome pathway *in vivo*..10

Appendix Table S1: Overview of the putative transcription factors identified by the selected OBP3 promoter region as bait in the yeast 1-hybrid screening assay...11

Appendix Table S2: Overview of putative prey proteins identified by OBP3 as bait in the yeast 2-hybrid screening assay...12

Appendix Table S3. The primers used in vector construction and mutant analysis......................13

Appendix Table S4. Gene-specific primers used in the qPCR analysis..14

Appendix Table S5. Gene-specific probes used in the EMSA experiments.................................16

Appendix Table S6. Gene-specific primers used in the ChIP-qPCR experiments.......................17

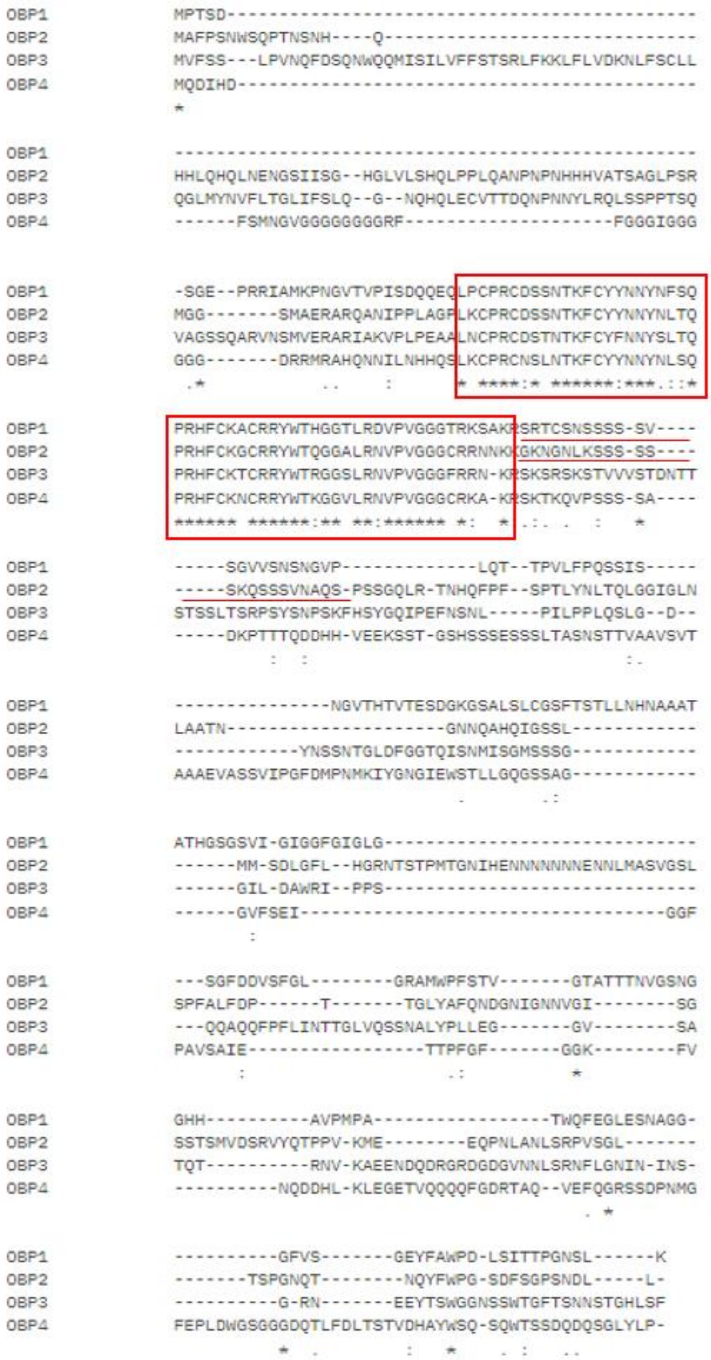

Appendix Figure S1: Alignment analysis of Arabidopsis OBP subgroup proteins.

Multiple sequence alignment of the amino acid sequence analysis among Arabidopsis OBP1-4 proteins were performed using ClustalW2 software (https://www.ebi.ac.uk/Tools/msa/clustalw2/). The Dof domain is boxed, and amino acids conserved among all the OBPs in the Dof domain are indicated by an asterisk. Serine-rich domains in OBP1 and OBP2 are under red solid line.

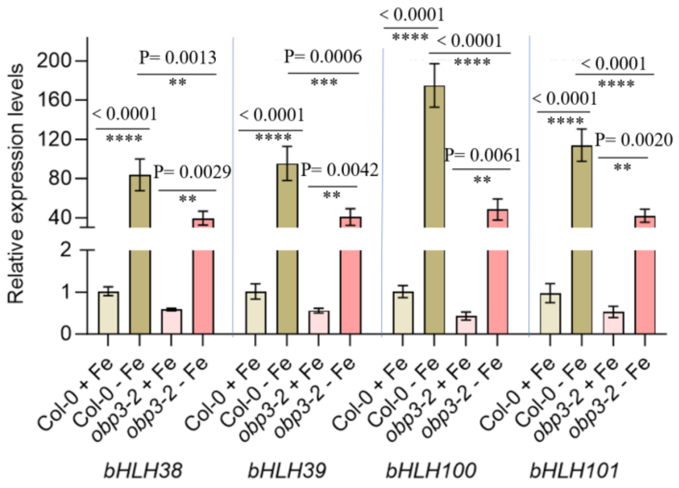

Appendix Figure S2: qPCR analysis of bHLH1b genes expression level.

In the background of the *obp3-2* mutant, iron deficiency (−Fe) induced upregulation of *bHLH38*, *bHLH39, bHLH100* and *bHLH101* gene expression. RT-qPCR analysis was conducted. The error bar represents SD (n = four independent pools, each composed of ten whole 7-day-old seedlings). Data were analyzed by two-tailed Student's *t*-test. Asterisk denotes significant differences (**$p < 0.01$, ***$p < 0.001$, ****$p < 0.0001$; Student's *t*-test). Exact P values are provided.

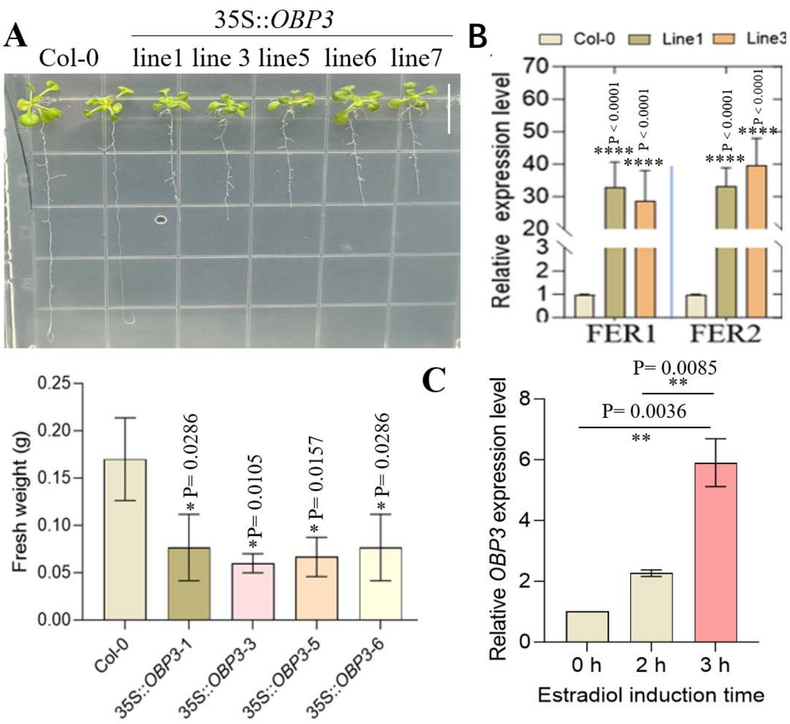

Appendix Figure S3: Analysis of root phenotype and expression level of *FER* genes in plants with constitutive overexpression of *OBP3*.

(A) Analysis of root phenotypes and the fresh weight in 9-day-old Col-0 and constitutive overexpression of *OBP3* plants. Bar indicates 1cm. Error bar represents SD (n = 3). (B) The relative expression of *FER1* and *FER2* in the 7 days old seedlings of wild-type and 35S::*OBP3* transgenic plants. The gene expression level of each gene in wild-type plants is set to 1. The data represents average ± SD for three independent experiments. (C) Estradiol treatment can up-regulate the level of *OBP3* gene expression in the pER8::*OBP3* transgenic plants. Detection of up-regulated expression of *OBP3* gene after 30 µM DEX induction for 0-3 h in the inducible transgenic plants. Error bar represents SD (n = 3). Data were analyzed by two-tailed Student's *t*-test. Asterisk denotes significant differences (**p* < 0.05, ****p* < 0.001, *****p* < 0.0001; Student's *t*-test). Exact P values are provided.

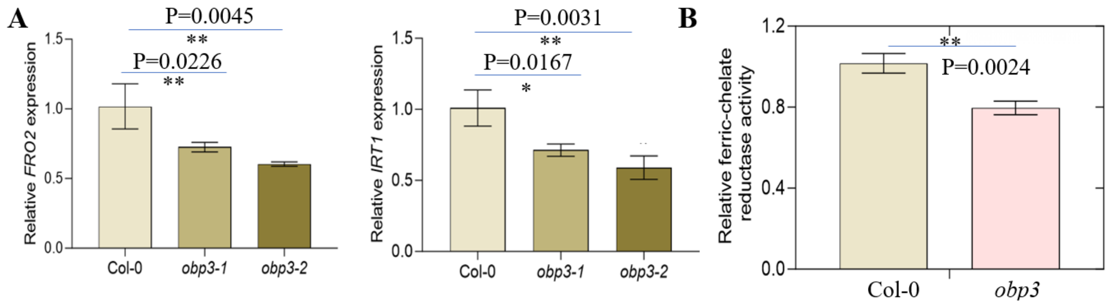

Appendix Figure S4: The gene expression levels of FRO2 and IRT1 and the activity of ferric-chelate reductase in Col-0 and *obp3* mutant root.

(A) RT-qPCR analysis *FRO2* and *IRT1* genes expression levels under the *obp3* mutants' background. Star indicates significant difference by students' *t*-test. Error bar represents SD (n = 3). (B) Detection of Ferric-chelate reductase activity of the wild type and *obp3* mutants' roots. The assay was performed on 10 pooled plant roots. The ferric-chelate reductase in Col-0 wild-type plants under +Fe is set to 1. Values are means ± SD. Data were analyzed by two-tailed Student's *t*-test. Asterisk denotes significant differences (*$p < 0.05$, **$p < 0.01$; Student's *t*-test). Exact P values are provided.

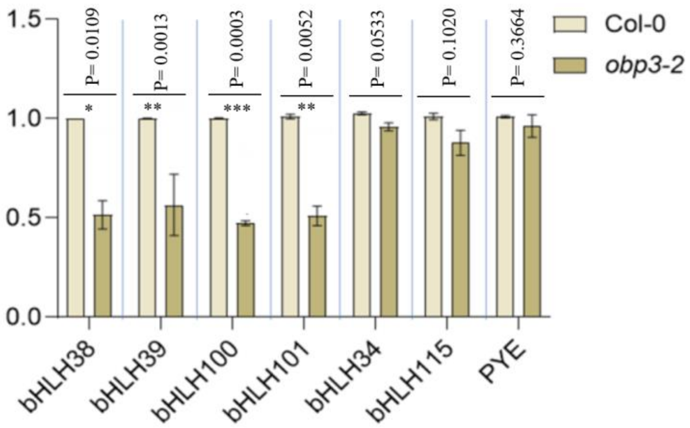

Appendix Figure S5: Relative expression levels of iron deficiency related genes in Col-0 WT and *obp3-2* mutant.

The assay was performed on 10 pooled plants. Two independent experiments were conducted. The relative expression of each gene in Col-0 background served as the respective control. Values are given as mean ± SD, n = 3. Data were analyzed by two-tailed Student's *t*-test. Asterisk denotes significant differences (*$p < 0.05$, **$p < 0.01$, ***$p < 0.001$; Student's *t*-test). Exact P values are provided.

DNA Alignment (Optimized Region)

Optimized	1	ATGGTTTTTAGTTCACTACCCGTAAATCAATTCGACTCCCAAAACTGGCAACAGATGATT
Original	1	ATGGTTTTCTCATCTCTTCCAGTGAATCAGTTCGATTCCCAAAATTGGCAGCAGATGATT
Optimized	61	AGCATTCTGGTGTTCTTCTCTACGAGCCGCCTGTTCAAGAAACTGTTTCTTGTCGATAAA
Original	61	TCAATTTTGGTCTTTTTTTCTACTTCAAGACTTTTTAAAAAATTATTCTTAGTTGATAAA
Optimized	121	AATCTGTTCTCCTGCCTGCTGCAGGGGCTGATGTATAACGTGTTCCTGACGGGTCTGATT
Original	121	AACCTTTTTTCTTGTCTTCTCCAAGGGCTTATGTATAATGTTTTTCTTACAGGATTAATT
Optimized	181	TTTTCTTTGCAGGGCAACCAGCATCAGCTAGAGTGCGTTACTACCGACCAGAACCCGAAT
Original	181	TTCTCTTTGCAAGGGAACCAACATCAGCTAGAATGTGTCACAACTGACCAGAACCCTAAT
Optimized	241	AATTACCTGCGTCAGCTGAGCTCTCCGCCAACGAGCCAAGTTGCGGGTAGCAGCCAAGCC
Original	241	AATTACTTACGGCAGCTCTCATCACCACCGACTTCTCAGGTTGCAGGTTCGAGTCAAGCT
Optimized	301	CGTGTTAATTCCATGGTGGAACGTGCACGCATCGCGAAGGTGCCGTTGCCGGAAGCGGCG
Original	301	AGAGTGAATTCAATGGTGGAACGTGCTCGGATCGCAAAAGTCCCATTGCCTGAAGCAGCT
Optimized	361	CTGAATTGCCCGCGTTGTGATTCCACGAACACCAAATTCTGCTACTTCAACAACTACAGC
Original	361	CTAAATTGCCCTAGATGTGACTCAACCAATACTAAGTTCTGTTACTTCAATAACTATAGC
Optimized	421	TTGACCCAGCCGCGCCACTTTTGCAAAACCTGTCGTCGTTACTGGACCCGCGGTGGATCT
Original	421	CTTACTCAACCTCGCCATTTCTGCAAAACATGTCGTCGCTATTGGACACGTGGCGGTTCC
Optimized	481	CTGCGCAACGTGCCTGTTGGTGGCGGCTTTCGTCGCAACAAACGCAGCAAGTCGCGCAGC
Original	481	TTGAGGAATGTTCCTGTTGGAGGAGGCTTTAGGAGGAACAAGAGAAGCAAATCCAGATCG
Optimized	541	AAGAGCACCGTTGTTGTCAGCACTGACAACACCACAAGCACTTCGTCCCTGACCAGCCGT
Original	541	AAATCTACGGTCGTGGTCTCGACTGATAATACTACTAGTACTTCATCACTTACTTCTCGC
Optimized	601	CCGTCTTACAGCAATCCGTCAAAGTTCCACAGCTACGGCCAGATTCCGGAGTTCAATTCT
Original	601	CCAAGTTACTCAAACCCTAGCAAGTTTCATAGCTACGGTCAAATCCCGGAGTTTAATTCC
Optimized	661	AACTTGCCGATCCTCCCACCGCTGCAAAGCTTGGGCGATTATAATTCCTCGAACACTGGC
Original	661	AACTTGCCCATCTTGCCTCCTCTCCAAAGCCTTGGAGATTACAATTCAAGCAACACTGGA
Optimized	721	TTAGACTTTGGTGGCACCCAAATCAGCAACATGATCTCCGGCATGAGCAGCAGTGGCGGT
Original	721	TTAGATTTTGGTGGAACTCAAATAAGCAACATGATAAGTGGTATGAGTTCTAGTGGTGGG
Optimized	781	ATTCTGGACGCTTGGCGTATCCCGCCGAGCCAACAAGCCCAGCAGTTTCCGTTTCTTATC
Original	781	ATCTTGGATGCATGGAGAATACCTCCATCACAACAAGCTCAGCAATTCCCTTTCTTGATC
Optimized	841	AACACGACCGGTTTGGTGCAGAGCTCGAACGCGCTGTATCCGTTACTGGAGGGTGGCGTC
Original	841	AACACTACCGGATTGGTGCAATCTTCAAACGCGTTATATCCATTACTAGAAGGCGGGGTT
Optimized	901	AGCGCAACCCAAACCCGTAATGTGAAGGCTGAAGAGAATGATCAGGATAGAGGTCGTGAT
Original	901	AGCGCCACGCAAACAAGAAATGTGAAGGCGGAAGAGAATGATCAGGATCGGGGTAGGGAT
Optimized	961	GGTGACGGCGTTAACAACCTCTCCCGTAACTTTTTGGGTAACATCAATATTAACTCCGGC
Original	961	GGGGATGGAGTGAATAACTTATCAAGAAACTTTTTGGGTAATATCAACATAAACTCAGGC
Optimized	1021	CGTAATGAGGAGTATACCAGCTGGGGTGGTAACTCCTCATGGACCGGATTCACCAGTAAC
Original	1021	AGGAACGAGGAATACACATCATGGGGAGGTAACAGTTCTTGGACCGGTTTCACCTCCAAC
Optimized	1081	AATTCTACGGGTCATTTGAGCTTTTAA
Original	1081	AACTCAACAGGCCATCTCTCATTCTAA

Appendix Figure S6: Codon optimization and *E. coli* bacterial expression of OBP3 protein.

We used the GenScript GenSmart codon optimization tool (https://www.genscript.com/ gensmart-free-gene-codon-optimization.html) to optimize the *OBP3* CDS sequence. Using the pMAL-C5x and pET-30a vectors and *E. coli* expression system to express the OBP3 protein. The induction condition is 0.3 mM IPTG at 28 °C for 4 h. SDS-PAGE gel of purified recombinant OBP3 protein. Purification of OBP3 protein using the TaKaRa Capturem™ His-Tagged Purification Kit according to the manufacturer’s instructions.

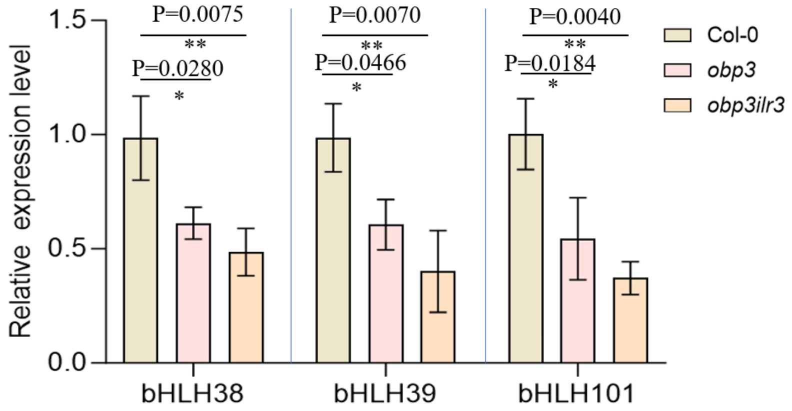

Appendix Figure S7: The *bHLH38*, *bHLH39*, *bHLH101* expression levels in Col-0, *obp3* single and *obp3ilr3* double mutant.

qPCR analysis was conducted on *bHLH38*, *bHLH39*, *bHLH101* expression levels in Col-0, *obp3* single and *obp3ilr3* double mutant backgrounds. Error bar represents SD (n = 3). Data were analyzed by two-tailed Student's *t*-test. Asterisk denotes significant differences (*p < 0.05, **p < 0.01; Student's *t*-test). Exact P values are provided.

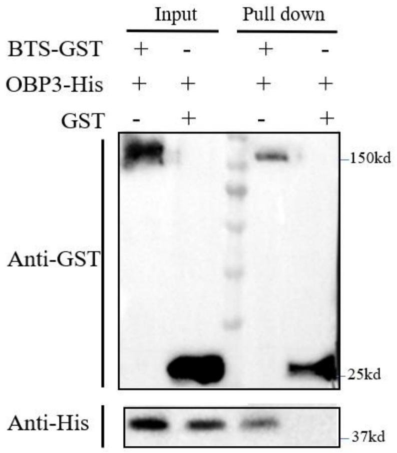

Appendix Figure S8: The direct interaction between OBP3 and BTS was also conducted by GST pull-down assay.

The GST-BTS fusion protein and His-OBP3 fusion protein were purified using glutathione beads (GE Healthcare), and Ni-NTA agarose (GE Healthcare). 2 μg samples of BTS-GST-bound glutathione beads were incubated with 2 μg His-OBP3 in binding buffer containing 20 mm Tris-HCl, pH7.5 and 200 mm NaCl at 4°C for 2 h. Wash the beads three times with washing buffers (20mM Tris-HCl, pH7.5 and 200mM NaCl). Protein eluted from beads was loaded onto 12% SDS-PAGE gel by boiling in a 2 × sampling buffer of 50 μL and eluted by anti-HIS (GenScript, A00186) or anti-GST (GenScript, A00865-100) for WB analysis.

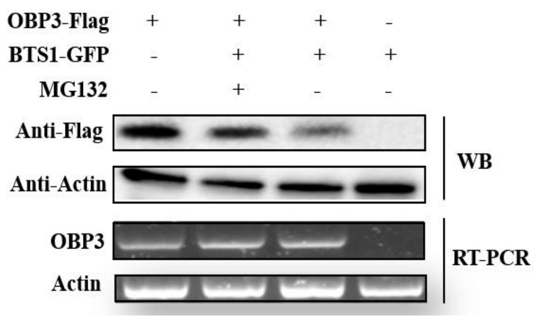

Appendix Figure S9: BTS promotes OBP3 degradation via the 26S proteasome pathway *in vivo*. To determine whether BTS-mediated ubiquitination triggers OBP3 degradation, we co-transfected BTS-GFP and Flag-OBP3 in Arabidopsis protoplasts. We observed a significant decrease in Flag-OBP3 protein abundance (not mRNA level) when in the presence of BTS-GFP; Flag-OBP3 degradation was recovered when protoplasts were treated with 50 µM MG132 (a 26S proteasome inhibitor). The protein abundance of OBP3-Flag was detected by immunoblotting using anti-Flag antibody. ACTIN was used as an internal control. OBP3 transcript level was measured by RT-PCR, and ACTIN was used as the internal control

Appendix Table S1: Overview of the putative transcription factors identified by the selected OBP3 promoter region as bait in the yeast 1-hybrid screening assay.

Gene ID	Family	Definition	Number of positive clones
At5g54680	bHLH	ILR3/ bHLH105	4
At1g51070	bHLH	bHLH115	2
At3g47640	bHLH	PYE	1
At3g55370	Dof	OBP3	2

Appendix Table S2: Overview of putative prey proteins identified by OBP3 as bait in the yeast 2-hybrid screening assay.

No.	Protein Names/Gene Names	Match
1	type one protein phosphatase 1	240/241(99%)
2		236/239(99%)
3	EXORDIUM like 3	108/108(100%)
5	SU(VAR)3-9 homolog 9	245/245(100%)
6	regulatory particle non-ATPase 13	237/239(99%)
7	\	
8	bHLH family transcription factor ILR3	116/116(100%)
9	EST gb|ATTS0956 comes from this gene	224/224(100%)
10	P-loop containing nucleoside triphosphate hydrolases superfamily protein	183/183(100%)
15	2Fe-2S ferredoxin-like superfamily protein	148/148(100%)
51		148/148(100%)
90		147/148(99%)
16	Crystal structure of photosynthetic A4 isoform glyceraldehyde-3-phosphate dehydrogenase complexed with NAD, from Arabidopsis thaliana	165/171(96%)
18	nuclear factor Y, subunit C4	143/143(100%)
19	Aldolase-type TIM barrel family protein	260/260(100%)
21	BRUTUS (BTS)	143/143(100%)
24	Leucine-rich repeat (LRR) family protein	218/218(100%)
25		218/218(101%)
26	2Fe-2S ferredoxin-like superfamily protein	148/148(100%)
27	hypothetical protein AALP_AA1G306700	142/142(100%)
28		142/142(100%)
29	lipid transfer protein; glossy1 homolog	139/139(100%)
30	aldehyde dehydrogenase 10A9	111/111(100%)
31	A. thaliana serine hydroxymethyltransferase isoform 4 (AtSHMT4) in complex with methotrexate	159/159(100%)
32	AT4g38970/F19H22_70	197/197(100%)
33	flavin-monooxygenase glucosinolate S-oxygenase 5	172/172(100%)
34	putative aspartic proteinase	103/103(100%)
35	Oxygen-dependent coproporphyrinogen III oxidase superfamily	184/185(99%)
36	zinc finger/BTB domain protein	136/137(99%)
37	PSI type III chlorophyll a/b-binding protein	190/190(100%)
38	peroxidase ATP4a, partial	198/198(100%)
39	SEUSS transcriptional co-regulator	256/257(99%)
40	AT1G20620	263/277(95%)

Appendix Table S3. The primers used in vector construction and mutant analysis.

**Transgenes**	**Primers (Sequence 5'-3')**
Clone *OBP3* cDNA	5'-ATGGTTTTCTCATCTCTTCCAGTGA-3′[a]
	5'-TTAGAATGAGAGATGGCCTGTTGA-3′[a]
pCAMBIA1300::*OBP3*	5'- CTGCAGTATCAAACTATAGATTTCGTGG -3′
	5'- GGGCCCCTTTGAATCTCTTACTTGTATCCTTT-3′
Double 35S::*OBP3*-HA	5'- GGATCC ATGGTTTTCTCATCTCTTCCAGTGA -3′
	5'-CTAGAA GAATGAGAGATGGCCTGTTGA -3′
pER8::*OBP3*	5'- ACTAGT ATGGTTTTCTCATCTCTTCCAGTGA-3′
	5'- CTCGAG GAATGAGAGATGGCCTGTTGA -3′
pHB::*OBP3*-GFP	5'- ACTAGT ATGGTTTTCTCATCTCTTCCAGTGA -3′
	5'- GAGCTC GAATGAGAGATGGCCTGTTGA -3′
pHB::*OBP3*-myc	5'- ACTAGT ATGGTTTTCTCATCTCTTCCAGTGA -3′
	5'- GAGCTC GAATGAGAGATGGCCTGTTGA -3′
proOBP3-OBP3-myc	5'- CTGCAG TATCAAACTATAGATTTCGTGG -3′
	5'- GAGCTC TACTGCCAAAGTAATAGTTGTCGG -3′
pET30a::*OBP3*	5'- GAATTC ATGAGAGCTGGTTTAAGTACGAT -3′
	5'- GGATCC TACTGCCAAAGTAATAGTTGTCG -3′
ILR3-GFP	5'- ACTAGT ATGGTGTCAC CCGAAAACGC-3′
	5'- GAGCTC TTAAGCAACAGGAGGACGAAGG-3′
obp3-1 (SALK_111615)	LP 5'- CCCTATGTCATGTTGTGATCG-3′
	RP 5'-CAACTCAACAGGCCATCTCTC-3′
obp3-2 (WiscDsLox377-380K7)	LP 5'-TCCGACCAATGTTTCAAACTC-3′
	RP 5'-TGAGTTGTTGGAGGTGAAACC-3′
Ilr3-2 (SALK_004997)	LP 5'-GAATTCACTAGGTTAATGCCCTG-3′
	LP 5'-TGCTAAGGTCAAACCATCCAC-3′

Restriction digestion sites are underlined.

a: Primers designed according to the 5'/3'-UTR for cloning *OBP3* gene cDNA.

Appendix Table S4. Gene-specific primers used in the qPCR analysis.

Genes	Primers (Sequence 5’-3’)
ACT2	5’- TCAGATGCCCAGAAGTCTTGTT -3’
	5’- CCGTACAGATCCTTCCTGATATC -3’
TUB2	5’- GAGCCTTACAACGCTACTCTGTC -3'
	5’- ACACCAGACATAGTAGCAGAAA -3'
OBP3	5'-GCCTTACTCAACCTCGCCAT-3'
	5'- TCGAGACCACGACCGTAGAT-3'
ILR3	5'-CGACGGGACAGGTTGAATGA-3'
	5'- GCAGCCTTGTCTGTTTTGGG-3'
bHLH038	5'- TGACCGACGCAAGAAGATCA-3'
	5'- GCCTCTTCACTTGCTGTTGC-3'
bHLH039	5'-CAATCCGGTGGTCGTCAAGA-3'
	5'- AGGCAGGAAGACATGAACGG -3'
bHLH100	5'- ACCGACGACGTATCCAACAC-3'
	5'- AGCTTCTTCATCACCACGGG -3'
bHLH101	5'-GCGAAAATCG CTACTCATAT GGAG-3'
	5'-GAACTGTTCTTGACTCGAGCTGCTA-3'
FER1	5'-AAGAAGATCGAGGAGCGGTG-3'
	5'-AGAGGCAAGAGAGCACGAAG-3'
FER2	5'-GCCACAAGTTCTCCGACGAT-3'
	5'-TTGGCGAAACCTTTCAAGCC-3'
NAS4	5'-CGTTGTGTTCTTGGCTGCTC-3'
	5'-CAGCATGAGCAAAGCACCAG-3'
FRD3	5'-CCTCTCTTTGCCACTGTCGT-3'
	5'-GCACCGATGATCCCTAGACG-3'
bHLH115	5'-AGCAAGAGATGAAGCGCAGA -3'
	5'-TCATCACGCAGCTCGTTCTT-3'
bHLH121/URI	5'-TCCCAGCTCCATGTCCTACA -3'
	5'-GAGAACGGTTACCGGGGTTC-3'

FIT1/bHLH029	5'-CCGATATCGCGGGTCTTGAA -3'
	5'-ACCGCGAAAAGGTTGAGTCT-3'
FRO2	5'-GCAAGCGAAGCTGGAATCAG -3'
	5'-AATCCCATTGCCGGTAGCAA-3'
PYE	5'-CGCAGCAGCCTTTGATGTTT-3'
	5'-GTAGCCGAGAAGACCACGAG-3'

Appendix Table S5. Gene-specific probes used in the EMSA experiments.

Genes	Probes (Sequence 5'-3')
bHLH038-c	5'- TAGAAAAGTTAATAACAAAGCAAATAA -3'
	5'- TTATTTGCTTTGTTATTAACTTTTCTA -3'
bHLH039-d	5'- AAGATTAATACCAAACGATACCA -3'
	5'- AAACGAGTGATAACAAAAAATGT -3'
bHLH100-g	5'-GCTATTTTGATTTGTTATAATTCAAG-3'
	5'-CTTGAATTATAACAAATCAAAATAGC-3'
bHLH101-k	5'-ACCATGAGAATTAGATAACAATTAATCA-3'
	5'-TGATTAATTGTTATCTAATTCTCATGGT-3'

Appendix Table S6. Gene-specific primers used in the ChIP-qPCR experiments.

Genes	Primers (Sequence 5'-3')
bHLH038-a	5'-TTTACATATATTCCTTGTTTGGTT -3'
	5'- AACAAAAAAGAAATCAGTTTTT -3'
bHLH038-b	5'- AAGATTAATACCAAACGATACCA -3'
	5'- TGTGTGTGAGTGTGACTGTGTGTC -3'
bHLH038-c	5'- ATCAAAGAAAGCTATAGAGAAGC-3'
	5'- TGCTGGCATATTTTGAAGATCGAGGG -3'
bHLH039-d	5'- GATAATAATTTCCGGAAAACCTATT -3'
	5'- TGAGATGCGTTGGTTCAAAAATA -3'
bHLH039-e	5'- CAAAAAAATGTAGCGATTGAAG -3'
	5'- CGTGTCGTTGTGTTTAGGTAATAG -3'
bHLH039-f	5'- AATAAAACAAAAATAGTGATTAT -3'
	5'- GTTGATTGCATATTTAATTTTAGG -3'
bHLH100-g	5'- CAAAAAAATGTAGCGATTGAAG -3'
	5'- CGTGTCGTTGTGTTTAGGTAATAG -3'
bHLH100-h	5'- AATAAAACAAAAATAGTGATTAT -3'
	5'- GTTGATTGCATATTTAATTTTAGG -3'
bHLH039-i	5'- CAAAAAAATGTAGCGATTGAAG -3'
	5'- CGTGTCGTTGTGTTTAGGTAATAG -3'
bHLH039-j	5'- AATAAAACAAAAATAGTGATTAT -3'
	5'- GTTGATTGCATATTTAATTTTAGG -3'
bHLH039-k	5'- CAAAAAAATGTAGCGATTGAAG -3'
	5'- CGTGTCGTTGTGTTTAGGTAATAG -3'
bHLH039-l	5'- AATAAAACAAAAATAGTGATTAT -3'
	5'- GTTGATTGCATATTTAATTTTAGG -3'
