# Supplementary figures and images for: The transcription factor Dof3.6/OBP3 regulates iron homeostasis in Arabidopsis
Source: EMBO J. 2024 Nov 13;44(1):251–68. doi: 10.1038/s44318-024-00304-0 (PMC11696086; doi:10.1038/s44318-024-00304-0)

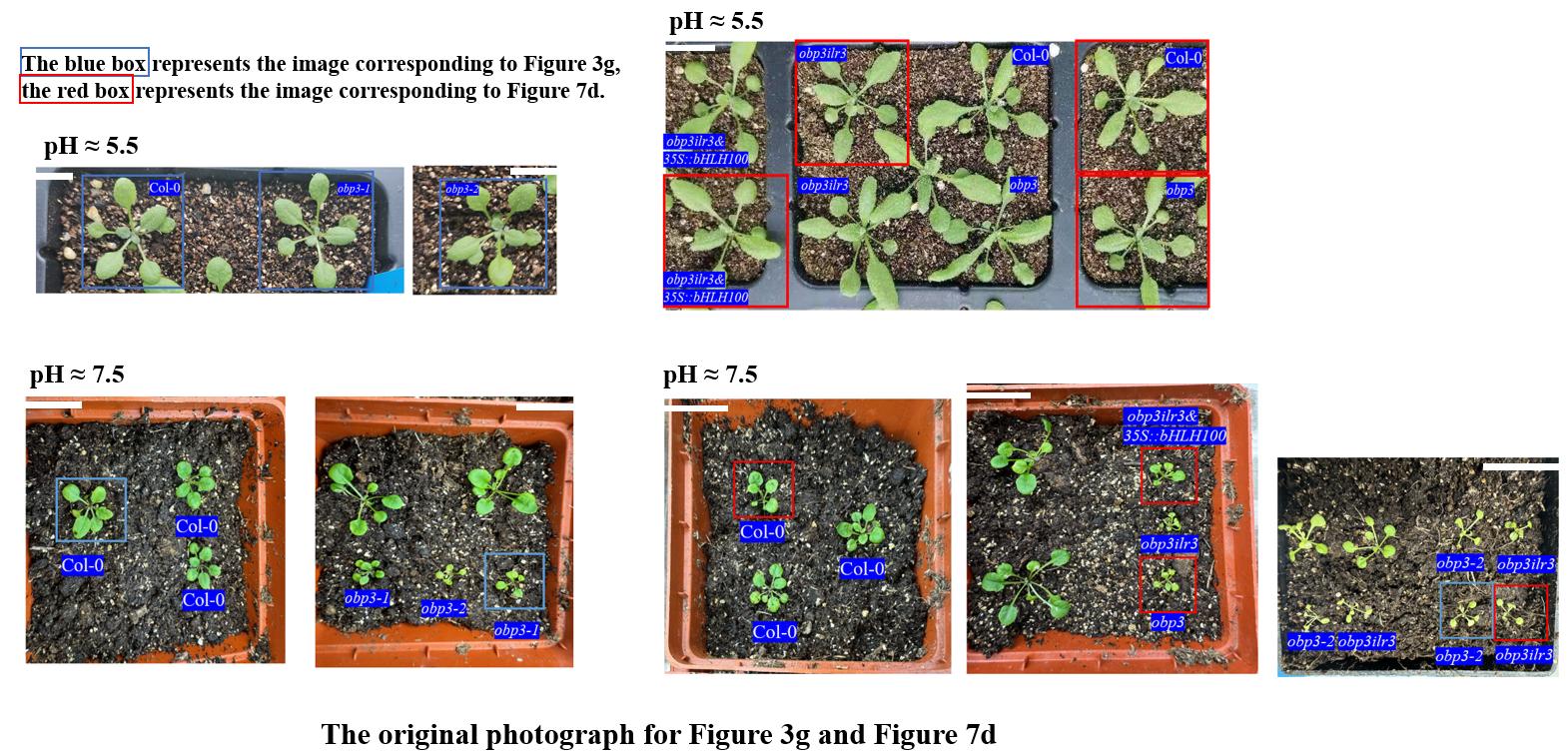

Supplement: Supplementary file 12 — Source data Fig. 3g and 7d [file 44318_2024_304_MOESM12_ESM.jpg]
